# Patient experience with pulmonary hypertension in Spain

**DOI:** 10.1186/s13023-025-03752-x

**Published:** 2025-05-20

**Authors:** José Joaquín Mira, Daniel García Torres, Manel Santiñá

**Affiliations:** 1 Departamento de Salud, Alicante-Sant Joan. FISABIO, Alicante, Spain; 2 Universidad Miguel Hernández de Elche, Elche, Spain; 3 Sociedad Española de Calidad Asistencial (SECA), Oviedo, Spain; 4 Institut d’Investigacions Biomèdiques August Pi i Sunyer (IDIBAPS), Barcelona, Spain

**Keywords:** Pulmonary arterial hypertension, Patient experience, Patient support, Disease management, Quality of life

## Abstract

**Background:**

This study explores the experiences of patients with pulmonary arterial hypertension (PAH) in Spain, focusing on the impact of patient support programs on disease management and quality of life. This study aimed to evaluate the effectiveness of patient support programs for PAH in improving disease management and quality of life of patients with PAH.

**Methods:**

A mixed study was carried out, applying qualitative and quantitative techniques with the aim of exploring and quantifying the experience of participants in support programs for PAH patients. It started with a integrate review of the literature which feed a qualitative phase where 14 patients (all women) diagnosed with PAH at least one year before were involved in focus groups. Subsequently, a quantitative phase was carried out, in which 36 patients, selected by non-probabilistic sampling, participated.

**Results:**

This study found that support programs substantially improve the quality of life and disease management in patients with PAH. In the qualitative phase, participants reported an improved perception of their ability to manage the disease, attributed to the emotional support and information provided. In the quantitative phase, considerable improvements were observed in several indicators of quality of life and disease management, corroborating the qualitative findings.

**Conclusions:**

Patient support programs are essential for improving disease management and the quality of life of patients with PAH. However, the findings highlight a gap in access to emotional and educational support within the healthcare system.

**Supplementary Information:**

The online version contains supplementary material available at 10.1186/s13023-025-03752-x.

## Background

Pulmonary arterial hypertension (PAH) is an incurable pulmonary vascular disease characterized by increased pulmonary vascular resistance, leading to progressive right ventricular overload and dysfunction, elevated pulmonary pressure, and ultimately, right heart failure [1, 2]. Patients commonly present with dyspnea, fatigue, chest and abdominal pain, tachycardia, and loss of appetite. As the disease progresses, physical activity becomes increasingly difficult. PAH affects approximately 15–50 individuals per million and is more prevalent among women [3]. Over the past two decades, advancements in specific therapies have contributed to improved survival rates [4]. Early diagnosis and timely treatment have been associated with increased long-term survival [5].

One of the therapeutic options for PAH involves continuous intravenous drug administration using a permanent infusion pump, requiring strict adherence to self-care guidelines. This treatment is particularly indicated in severe cases where continuous prostacyclin infusion is necessary as a bridge to lung transplantation or when immediate and sustained therapeutic effects are required. Owing to the complexity of this therapy, patients must demonstrate the ability to adhere to stringent self-care protocols.

Common complications associated with this treatment include infections (both local site and catheter-related bloodstream infections), thrombosis, pump malfunction, adverse drug reactions (such as headache, flushing, nausea, diarrhea, and jaw pain), and self-care errors that may increase safety risks. To address these challenges, psycho-emotional support and educational interventions have been implemented to help patients manage self-care responsibilities, including drug administration, pump usage, hygiene practices, and potential complications. However, despite the anticipated benefits and positive reception of these support programs, their effectiveness has not yet been systematically evaluated.

As with other rare diseases, assessing patient experiences with PAH remains challenging owing to the dispersed nature of the patient population [6, 7]. Nevertheless, measuring patient-reported outcomes of health intervention is crucial for identifying inefficiencies, inadequacies, or redundancies in care processes and advancing integrated management strategies for chronic conditions [8]. In Spain, a free psycho-emotional support program was developed alongside infusion pump therapy, designed by the Foundation for the Development of Training and Research in Health Sciences. This program included support from both a nurse who assisted patients with self-care concerns (such as drug administration, pump usage, and other daily situations) and a psychologist who addressed the emotional and psychological impact of the disease and its treatment. While the program appeared to be well received by patients, no formal study had been conducted to assess patients’ experiences with this support system.

## Methods

### Study aim, design, and setting

This study aimed to evaluate the experiences of patients with PAH undergoing infusion pump therapy and their access to a psychoemotional support program to provide valuable insight into the impact of such interventions on patient well-being and disease management. This mixed-methods study employed both qualitative and quantitative research techniques to explore the experiences of patients with PAH participating in a support program and measure those experiences using a specifically designed instrument.

This study was conducted between October 2023 and February 2024. It involved a fully anonymous, non-interventional survey focusing on participants’ experiences. The principles of the Declaration of Helsinki were followed. Invitations and participant recruitment were managed by the collaborating patient associations. Confidentiality was strictly maintained, and participation was entirely voluntary at all stages. Participants were free to withdraw from the study at any time. They were informed about the voluntary nature of their involvement and the objectives of the study. Informed consent was obtained from all participants to uphold ethical standards and respect their rights. No personally identifiable data were collected, and no clinical interventions were performed. Therefore, in accordance with applicable regulations, approval from an ethics committee was not required.

**Fig. 1:**
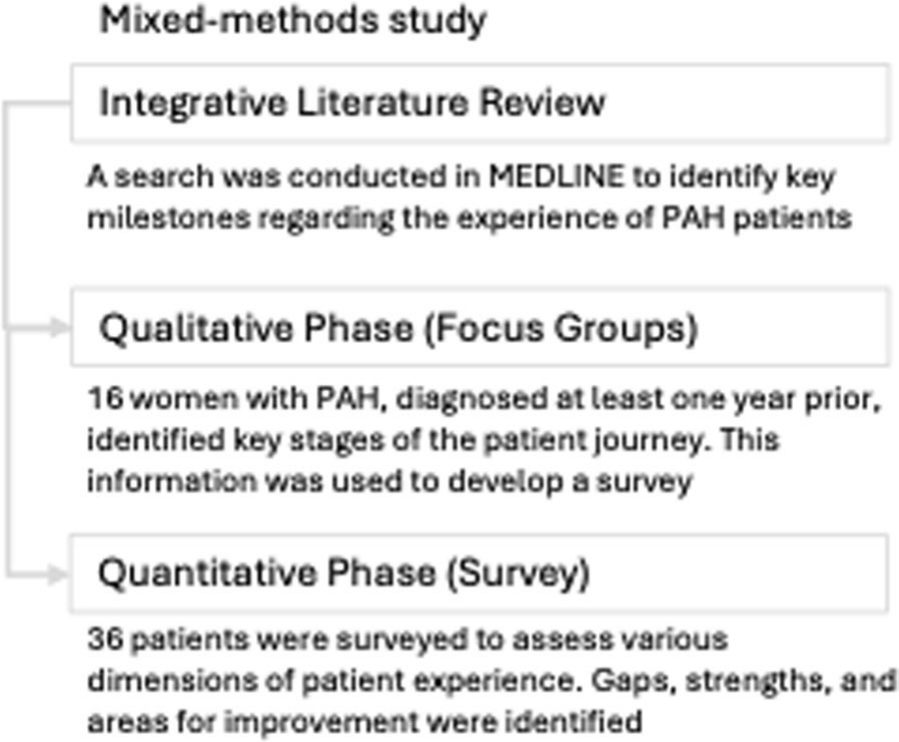
Study flowchart

The study was conducted in three key stages. Initially, an integrative literature review on PAH was conducted, with a focus on factors influencing therapeutic adherence and improvements in patient care quality. Subsequently, two focus groups were established involving patients affected by PAH to gather firsthand insights and relevant experiences. Finally, based on the data obtained from these focus groups, an initial version of a questionnaire was drafted to systematically assess patients’ experiences (Fig. 1).

### Integrative review steps

A comprehensive search of the MEDLINE database was conducted using clearly defined inclusion and exclusion criteria. Titles and abstracts were screened to identify relevant studies. Ryan’s tool was employed to manage all the collected data. Key details were recorded, focusing on aspects that categorized patients' experiences and the instruments used. In addition, the main findings were extracted and analyzed by two researchers, informing the development of guidelines for conducting the focus groups.

### Participants in phases two and three

The inherently low prevalence of PAH substantially limited the number of potential participants. Furthermore, the geographically dispersed residences of affected individuals posed further challenges in recruiting a larger sample. For each focus group, a minimum of eight participants (16 in total) diagnosed with PAH at least 1 year prior were included. Participants were recruited using the snowball technique from among members of three existing patient associations for this disease: Asociación Nacional de Hipertensión Pulmonar (National Pulmonary Hypertension Association, ANHP), Fundación contra la Hipertensión Pulmonar (Pulmonary Hypertension Foundation), and Hipertensión Pulmonar España Organización de Pacientes (Pulmonary Hypertension Spanish Patients Organization). The inclusion criteria were patients diagnosed with PAH at least 1 year previously, who were willing to participate in the study for at least 1 h, and provided informed consent. The exclusion criteria were as follows: minors, that is, individuals under 18 years of age, and those who are unwilling to participate voluntarily.

In the quantitative phase, a minimum of 30 patients were surveyed, representing a substantial effort given the rarity of the disease. Participants were members of the three patient associations collaborating in this study. Recruitment was performed using nonprobabilistic sampling. The three pulmonary arterial hypertension patient associations informed the members of the support program at the start of the survey. The inclusion and exclusion criteria were identical to those of the previous phase and included active follow-up in a support program.

### Support program

The psycho-emotional support program was voluntary and free of charge. Participation is open for as long as both the physician and the patient consider it beneficial. It was launched in 2020, with dedicated full-time staff assigned to its implementation.

### Study materials.

#### Integrative review

Before engaging with patients, a literature review on Patient-Repoted Experience Measures (PREMs), general patient experience, and satisfaction was conducted. The PubMed search engine within MEDLINE was used with the keywords Patient-Reported Experience Measures, Patient Experience, rare diseases, and PAH. The search was limited to publications from the last 10 years to identify relevant studies on PAH, its impact on patients and families, and any existing research assessing patient experiences.

### Focus groups

The research team, in consensus with ANHP representatives, developed a structured guide for the focus groups, which were conducted via Zoom teleconferencing. Three main questions and 11 clusters were established to guide discussions, with each session lasting no more than 45 min. Experienced facilitators lead the group discussions, ensuring the active participation of all attendees and comprehensive exploration of planned topics. Two research team members systematically coded participant responses. The collected data were organized and classified by the researchers into distinct units of analysis, using mutually exclusive categories that represented the patient's journey from pre-program enrollment through program initiation and ongoing follow-up**.**

### Survey

A questionnaire was developed based on information gathered from the focus groups, organized into categories reflecting the most relevant patient concerns. Each category was carefully considered during the creation of the survey items. Through successive iterations, both wordings and themes to be explored were enhanced for clarity and comprehensiveness. The instrument was reviewed and improved by the research team and focus group participants to ensure face and content validity. The final version of the questionnaire contained 21 items, covering all priority dimensions identified by patients. Legibility was assessed in four patients. A two-month response period was defined to allow participants to complete the survey. Six reminders were used in this study. Participants did not receive any compensation for their participation.

### Statistical analysis

For qualitative analysis and interpretation of data in phases two and three, thematic analysis was applied to gain a deeper and more detailed understanding of patients' experiences and perceptions. At the same time, descriptive statistics were used to summarize and interpret the data quantitatively.

## Results

### Literature review

A total of 62 scientific publications were initially considered; ultimately, 20 studies were selected that included information about the guide of treatment for patients with PAH, patient quality of life in rare diseases, or aspects related to patient experiences.

### Focus groups

A total of 16 female participants diagnosed with PAH participated in the study, with ages ranging from 40 to 68 years. Two participants had been enrolled in the support program before 2020, and all had been diagnosed with the condition for at least 10 years. Each association provided three participants. In total, two focus groups with nine participants each were organized; however, owing to absences, only eight participants attended the session.

The 'Patient Journey' of the participants in the support program was analyzed, identifying three key phases: pre-enrollment experiences, the process of joining the program, and the subsequent follow-up.

Before their incorporation into the PAH support program, the patients reported being diagnosed after experiencing critical situations and urgent hospitalization. Direct instructions by program professionals in the hospital on the use of the infusion pump and medication were mentioned. However, there were cases where the primary care physician had limited knowledge of the disease and the program and situations where patients or their families had to manage complications on their own. Information about the program was often received at the hospital, and some expressed the usefulness of the after-hour support provided by program professionals.

Once enrolled, participants emphasized the importance of receiving detailed information and educational materials on infusion pump use, pain management, and infection prevention. They mentioned variability in the pain experience and pain management strategies, such as the use of paracetamol and anti-inflammatory drugs. The duration between the point changes and psychological management of pain anticipation was also noted. Some expressed the need for additional support in managing pediatric patients, especially at point changes, and the usefulness of direct contact with program professionals to facilitate adaptation.

During follow-up within the program, several areas for improvement were identified: the need for more training of healthcare personnel on the disease and treatment, the lack of nurses specializing in the management of PAH, and the need for specific training in schools to support pediatric patients. In addition, the importance of providing psychological support proactively was emphasized, and the need to empower patients to take a more active role in their care and reduce dependence on specific advice from professionals was highlighted.

### Questionnaire

A total of 36 patients with PAH participated in this study (Table 1).

**Table 1:** Distribution of participants in the PREM study

	N	%
Sex		
Females	29	80.6
Males	7	19.4
Age		
30–50	6	16.7
51–75	29	80.5
> 75	1	2.8
Years since diagnosis		
< 5	9	25.0
6–10	5	13.9
> 10	12	33.3
Years in the program		
< 3	15	41.7
4–5	6	16.7
> 5	1	2.8

N = 36

*PREM* Patient-reported experience measures

Regarding patients' experiences with primary care professionals (P1), 26.5% of the respondents stated that they always received informed and appropriate care, while another 26.5% indicated that this only happened occasionally. Regarding coordination between the different levels of healthcare (P2), 45.7% of the patients reported that there was always good communication between professionals, suggesting effective collaboration in their care.

With respect to the knowledge and assistance provided by emergency departments (Q3), 25% of the respondents always had knowledgeable staff and adequate assistance, compared with 18.7% who rarely or never had this experience.

Regarding the information provided by hospital professionals about the disease and its treatment (Q4), 65.7% of the patients reported that they were always given all the necessary information, compared with 2.86% who never received it. With respect to the information provided by health center professionals (Q5), 9.09% of the respondents stated that they always received all the necessary information, whereas 39.39% never received it.

Regarding information on the patient support program at the hospital where treatment was indicated (Q6), 63.6% of patients stated that they were always informed about the program, compared with 24.2% who reported never receiving such information.

A total of 75.8% of patients reported that the hospital or service where they were instructed on treatment and infusion pump use (P7) connected them to the referral person for the PAH support program. However, 18.2% reported not receiving this referral. Furthermore, 52.9% had a preliminary meeting with their physician and nurse before joining the support program (Q8), whereas 44.1% did not. On the other hand, 33.3% of respondents felt insecure and had difficulty accessing information before entering the support program (Q9), while 22.2% did not experience such difficulties.

Table 2 shows the patients’ views regarding the process of information and contact with the support program for patients with PAH and the information provided for pump management.

**Table 2:** Participants' response to the information and contact process

Ask	N	Yes (%)	No (%)		Not Applicable (%)	
P10. The process of information and contact with the support program for patients with PAH was simple and quick	36	94.4	0.0		5.6	
Ask	N	Not Applicable (%)	Nothing (%)	Little (%)	Quite (%)	All (%)
P11. Once in the support program for patients with PAH, the nurse provided me with information about how to use the pump	36	2.8	2.8	2.8	19.4	72.2
P12. The patient support program staff provided me with information on drug administration and how to care for the insertion site	34	8.8	0.0	0.00	23.5	67.7

Most survey participants rated the support received through the patient support program positively. In response to question Q13, 80% stated that the staff provided them with useful information to prevent and address complications and side effects of the treatment. Regarding question Q14, 50% of the respondents indicated that thanks to the program, they avoided emergency room visits or long waits for medical consultations. In question Q15, 68.6% expressed feelings of being in control and were able to face problems related to their disease owing to the help provided by the program.

In the context of the support provided by professionals in the support program for patients with PAH (Q16), an overwhelming 91.7% of the patients stated that they always received the necessary help in the event of any doubt or complications. In emergencies related to their disease (Q17), more than half (58.3%) preferred to go to support program professionals rather than to the emergency department. In addition, a significant majority (63.9%) of respondents felt that without the support program, they would feel helpless and would be forced to resort more often to emergency services to resolve problems or complications (Q18).

Regarding the emotional assistance provided by the psychologist in the support program (Q19), a significant majority (51.6%) of the patients stated that this help enabled them to overcome the emotional impact of the disease. Regarding communication and coordination between professionals in the support program and those in the hospital (Q20), 58.3% perceived effective collaboration between the two. Finally, in relation to the care received in the hospital and the availability of contact in case of problems (Q21), 63.9% of the respondents felt that healthcare professionals were attentive to their needs and accessible for consultations about the disease or use of the pump.

Patients in the support program suggested some key areas for improvement: they highlighted the importance of psychological care, even if they did not use it frequently; proposed the inclusion of group psychotherapy to improve psychological care; requested more information about possible side effects before starting treatment; and expressed the need for more knowledge about all services offered by the program.

## Discussion

The focus group results reflected the heterogeneity of patient experiences before admission to the support program for patients with PAH. It was evident that the detection of the disease in primary care was late and that instruction on infusion pump management came mainly from the hospital setting. The lack of information regarding PAHs in primary care and emergency departments highlights the need for an initiated reference unit for rare diseases. In addition, it was identified as the main target for improving patients’ journeys between different levels of medical and nursing care [9]. The importance of the availability of psychological and technical support, both within the program and in emergencies, as essential components for effective disease management was established [10, 11].

Once enrolled in the support program, patients report variability in pain experiences and individual disease management strategies, suggesting that support should be further personalized [12], especially in areas such as pain and infection control [13]. In addition, patients also emphasized the importance of psychological preparation and ongoing support, which aligns with findings from other studies, underscoring the need for effective and accessible communication between patients and program professionals to enhance the treatment experience.

Regarding follow-up within the program, the findings emphasized the need for anticipatory psychological support and the promotion of patient autonomy. This suggests a shift towards a more comprehensive model of care, emphasizing self-management and minimizing dependence on individual advice from healthcare professionals.

The results reflect diverse experiences among patients in different health services, highlighting areas of potential improvement [14]. Variability in primary care and emergency departments presents a challenge for clinicians and managers. The positive perception of patients, when the coordination between services is correct, indicates other challenges in the healthcare system.

The information provided to patients about their disease varied considerably between hospitals and health centers [15]. In hospitals, most patients receive complete information when they receive assistance from the same professionals in rare disease departments, suggesting a high level of communication in these settings. Otherwise, a high percentage of patients reported not receiving all the necessary information, which again denoted a significant gap in communication between healthcare centers and levels of care, despite this being a critical area for ensuring that patients receive complete and effective information about their disease and treatment.

Most patients were adequately informed at the hospital, where they were prescribed treatment for the patient support program and were in contact with a referral person. However, there is a percentage who stated that they did not receive this information.

Most patients found the processing of information and contact with the patient support program simple and rapid. This indicates that the program was easily accessible to patients. However, once in contact with the program, a considerable proportion never had a preliminary meeting with the program physician or nurse, which may have prevented some patients from joining the program. In addition, before joining the program, one-third felt insecure and had difficulties resolving doubts about their disease, which underlines the importance of early and accessible support for patients with PAH.

Once enrolled in the support program for patients, most patients reported receiving all necessary information on infusion pump use and insertion site care from the nursing staff. These data accentuate the effectiveness of the program in providing essential education and support for therapy management, a critical aspect of treatment success and patient autonomy.

Another aspect that highlighted the importance of the program is its usefulness in helping patients prevent and manage the complications and side effects of the disease, which allowed half of the patients to avoid visits to the emergency room or unnecessary waits for medical consultations, demonstrating its effectiveness in improving personal health management and in providing a continuous avenue of support. In addition, most patients relied on the support received from PAH program professionals to resolve questions and complications, preferring this option to the emergency department.

Continuing with patient autonomy, the majority of patients felt in control and were able to cope with the challenges associated with their disease thanks to the support program for patients with PAH, which underscored the perceived effectiveness of the program in providing a safety net and improving patient autonomy in disease management.

Psychological support within the support program for patients with PAH was critical for more than half of the participants, who reported that it substantially helped them manage the emotional impact of their disease. However, a considerable percentage of patients did not perceive this benefit, indicating the need to evaluate and improve the provision of psychological support services within the program.

The findings of this study align with the recommendations outlined in the Guidelines for the Diagnosis and Treatment of PAH [16], which emphasizes the importance of a comprehensive approach to disease management. The reported improvement in the quality of life and self-management among patients participating in the support program reinforces the need to implement non-pharmacological interventions that complement standard medical treatment. In addition, the coordination between different levels of care identified as a key area for improvement in this study was consistent with the challenges outlined in the guidelines for optimizing disease management.

Coordination between the professionals in the support program for patients with PAH and hospitals proved effective, with most patients perceiving good communication and willingness to be contacted in cases of need. This indicates a level of functional integration between different levels of care benefiting patient management.

Support programs for patients with chronic and complex diseases have been developed in Spain because of their effectiveness in preventing exacerbations, complications, and unnecessary emergency department visits. However, these programs have not yet been fully integrated into the National Health System. Patient associations, in collaboration with clinicians, are working on implementing them for rare diseases such as PAH. Although progress has been made, further progress is needed to strengthen and expand these initiatives. Patients receiving support appeared to reduce the strain on hospital services by avoiding unnecessary emergency visits. Further research on the cost-effectiveness of these programs is essential, particularly for rare diseases, where resource allocation is more challenging than in more common conditions.

The main implication of these results for managers and healthcare policymakers lies in the positive effect of listening to patients, considering the patient’s journey as a whole^6^, and the support provided by specific units and programs based on the frequent approach, The role of rare disease-patient associations was reinforced in this study [17].

## Limitations

Most participants who received support from the patient support program were involved in patient associations, interactions, and communication with others, which might have impacted their views and experiences. As in studies focusing on rare diseases, the number of participants was limited [18]. Data on patients' treatment experiences were not compared with those of patients who were not enrolled in support programs, making it impossible to attribute their experiences solely to the support program.

## Conclusions

This study emphasizes the importance of improving early detection, training, and continuous support to address treatment challenges, as well as the need for effective communication, promotion of patient autonomy, and coordination between healthcare services to enhance the overall treatment experience. Support programs are essential for improving disease management and the quality of life of patients with PAH.

## Supplementary Information

Additional file 1.

Additional file 2.

## Data Availability

The datasets generated and/or analyzed during the current study are available in Open Science Framework (OSF), titled "Patient Experience with Pulmonary Hypertension in Spain", located at https://osf.io/h3e7v/.

## Abbreviations

PAH: Pulmonary arterial hypertension
ANHP: Asociación Nacional de Hipertensión Pulmonar
PREMs: Patient-reported experience measures
