# Supplementary material for: Patient experience with pulmonary hypertension in Spain
Source: Orphanet J Rare Dis. 2025 May 20;20:239. doi: 10.1186/s13023-025-03752-x (PMC12093829; doi:10.1186/s13023-025-03752-x)
Supplement: Supplementary file 1 — Additional file 1. [file 13023_2025_3752_MOESM1_ESM.docx]

Additional file 1. Participants' responses about the health care system in relation to their disease.

| Ask | N | Never (%) | Occasionally (%) | Sometimes (%) | Almost always (%) | Always (%) |
| --- | --- | --- | --- | --- | --- | --- |
| P1. The health professionals who treated me at my primary care center knew my disease, knew how to care for me, and answered my questions well. | 34 | 14.7 | 26.5 | 11.8 | 20.5 | 26.5 |
| P2. The professionals who treated me at my hospital, at my health center, and those who treated me at my referral center talked to each other and coordinated to make sure I received the correct diagnosis and treatment. | 35 | 14.3 | 2.9 | 14.3 | 22.9 | 45.6 |
| P3. When I went to the ER, they were knowledgeable about my condition and helped me with my practice problem. | 32 | 12.6 | 18.7 | 18.7 | 25.0 | 25.0 |
| P4. The professionals at the hospital gave me all the necessary information about my disease, the treatment, and its side effects. | 35 | 2.9 | 0.0 | 8.6 | 22.8 | 65.7 |
| P5. The professionals at the health center gave me all the necessary information about my disease, the treatment, and its side effects. | 33 | 39.4 | 12.1 | 12.1 | 27.3 | 9.1 |
| P6. At the hospital where I was prescribed the treatment and the use of the pump, I was informed of the patient support program that is available for this treatment. | 33 | 24.2 | 6.2 | 3.0 | 3.0 | 63.6 |
| P7. At the hospital/service where I was indicated for treatment and use of the pump, I was put in contact with the reference person for the support program for patients with PAH. | 33 | 18.2 | 6.1 | 0.0 | 0.0 | 75.7 |
| P8. I attended a meeting with my physician and the patient support program nurse before admission to the support program for patients with PAH. | 34 | 44.2 | 2.9 | 0.0 | 0.0 | 52.9 |
| P9. Before joining the patient support program, I felt insecure and had difficulty accessing someone who could answer my questions. | 36 | 22.2 | 13.9 | 22.2 | 8.4 | 33.3 |

PAH, pulmonary arterial hypertension; ER, emergency room
