# Supplementary material for: Patient experience with pulmonary hypertension in Spain
Source: Orphanet J Rare Dis. 2025 May 20;20:239. doi: 10.1186/s13023-025-03752-x (PMC12093829; doi:10.1186/s13023-025-03752-x)
Supplement: Supplementary file 2 — Additional file 2. [file 13023_2025_3752_MOESM2_ESM.docx]

Additional file 2. Participants' responses about the support program.

| Ask | N | Never (%) | Occasionally (%) | Sometimes (%) | Almost always (%) | Always (%) |
| --- | --- | --- | --- | --- | --- | --- |
| P13. The patient support program staff gave me useful information about typical complications of my disease, the side effects of treatment (inflammation, infection, pain...), and how to deal with them. | 35 | 2.9 | 0.0 | 0.0 | 17.1 | 80.0 |
| Q14. Thanks to the patient support program, I avoided having to go to the emergency room or having to wait to contact my physician when a consultation was possible. | 36 | 8.3 | 2.8 | 11.1 | 27.8 | 50.0 |
| P15. I have the feeling that I am in control and that I will be able to cope with the problems that arise from my disease thanks to the help I am given in the support program for patients with PAH. | 35 | 0.0 | 2.8 | 5.7 | 22.8 | 68.7 |
| P16. The professionals in the support program for patients with PAH who care for me help me when I have any questions or  complications as a result of my disease. | 36 | 8.3 | 0.0 | 0.0 | 0.0 | 91.7 |
| P17. If I have a problem related to my illness that requires urgent attention, I prefer to go to the professional in the support program rather than to the emergency room. | 36 | 11.1 | 0.0 | 13.9 | 16.7 | 58.3 |
| Q18. If I had not enrolled in the support program for patients with PAH, I would have felt helpless, and I would have had to go to the hospital more often to resolve problems or doubts. | 36 | 5.6 | 5.6 | 13.9 | 11.1 | 63.8 |
| P19. The help of the psychologist in the support program for patients with PAH helps me cope emotionally with the disease. | 31 | 32.5 | 0.0 | 3.0 | 12.9 | 51.6 |
| P20. I see that the professionals in the support program for patients with PAH communicated and coordinated with the professionals in my hospital. | 36 | 11.1 | 0.0 | 5.5 | 25.0 | 58.4 |
| P21. The health professionals who care for me at the hospital are looking out for me, and I can contact them if I have problems with any aspect of the disease or the pump. | 36 | 2.8 | 2.8 | 8.3 | 22.2 | 63.9 |

PAH, pulmonary arterial hypertension.
